# Source of Social Support and Caregiving Self-Efficacy on Caregiver Burden and Patient’s Quality of Life: A Path Analysis on Patients with Palliative Care Needs and Their Caregivers

**DOI:** 10.3390/ijerph17155457

**Published:** 2020-07-29

**Authors:** Doris Y. P. Leung, Helen Y. L. Chan, Patrick K. C. Chiu, Raymond S. K. Lo, Larry L. Y. Lee

**Affiliations:** 1 School of Nursing, The Hong Kong Polytechnic University, Hong Kong 999077, China; 2 The Nethersole School of Nursing, The Chinese University of Hong Kong, Hong Kong 999077, China; helencyl@cuhk.edu.hk; 3 Department of Medicine, Li Ka Shing Faculty of Medicine, The University of Hong Kong, Hong Kong 999077, China; chiukc@ha.org.hk; 4 Department of Palliative Medicine, Shatin Hospital, Hong Kong 999077, China; losk@ha.org.hk; 5 A & E Medicine Academic Unit, The Chinese University of Hong Kong, Hong Kong 999077, China; lly849@ha.org.hk

**Keywords:** caregiver burden, caregiving self-efficacy, quality of life, palliative care, sources of social support

## Abstract

Few studies have explored the inter-relationships of sources of social support and caregiving self-efficacy with caregiver burden and patient’s quality of life among patients with palliative care needs and their caregivers. This study tested the associations of two sources of social support (family and friends) and the mediating role of caregiving self-efficacy on caregiver burden and patient’s quality of life. A convenience sample of 225 patient–caregiver dyads recruited between September 2016 and May 2017 from three hospitals in Hong Kong was included in the current analysis. Results showed that the final model provided a satisfactory fit (SRMR = 0.070, R-RMSEA = 0.055 and R-CFI = 0.926) with the data, as good as the hypothesized model did (*p* = 0.326). Significant associations were detected. Family support had a significant negative indirect effect on caregiver burden and a significant positive indirect effect on patient’s quality of life through caregiving self-efficacy, whereas friend support had a significant positive direct effect on caregiver burden but a minimal effect, if any, on patient’s quality of life. These findings emphasized (1) the importance of caregiving self-efficacy in improving caregiver burden and patient’s quality of life and that (2) sources of social support may be an important dimension moderating the associations of caregiving self-efficacy with caregiver burden and patient’s quality of life.

## 1. Introduction

Palliative care emphasizes meeting the needs of patients to improve quality of life of both patients and their families facing problems associated with life-threatening illnesses [1]. World Health Organization (WHO, Geneva, Switzerland) recommends that palliative care should cover from the time of diagnosis, alongside potentially curative treatment, to disease progression and the end of life [2]. Globally, 40 million people are estimated to require palliative care each year, and most of them are cared for in their own homes [3]. The chronicity of these illnesses shifts the care from the hospital setting to the community, leading to an unprecedented dependence on family caregiver support. Many caregivers of patients with advanced or terminal illness are overburdened [4]. Providing care to patients can be stressful and is expected to worsen especially for patients who need palliative care because of the chronic nature of their diseases, lack of foresight about the time of its finalization, and emotional distress of the expected loss of the patient [5]. Caregivers themselves are also in need of support when facing the additional concurrent stress of significant role transitions and the responsibilities of managing patients’ needs, commonly resulting in caregiver burden [4]. This may negatively affect the family caregiver’s ability to provide quality care and may result in poor patient outcomes including poorer quality of life and distress exacerbation [6,7,8].

Caregiving could be beneficial despite being stressful. For example, caregivers who positively appraised caregiving situations might become stronger people and improve their communication skills [9], possibly leading to improved patient’s quality of life by providing better care. Caregiving self-efficacy is the perceived confidence in one’s ability to perform the tasks of caregiving that are amenable to change [10]. Several studies have demonstrated the important role of caregiving self-efficacy in reducing caregiver burden and improving patient outcomes in palliative care. Caregiving self-efficacy is related to reduced burden and anxiety and increased positive aspects of caregiving and self-care behaviors among caregivers of patients with advanced chronic diseases [11,12,13,14,15]. Caregivers with high levels of self-efficacy are generally considered to be proactive in self-care and to complete activities that help to improve health, while caregivers with low self-efficacy are likely to experience negative outcomes such as anxiety and depression [16]. Research has reported that higher caregiver self-efficacy is associated with a better experience in palliative cancer patients, including having more energy, feeling less ill, and spending less time in bed [17], suggesting that caregiver self-efficacy is associated with patient’s quality of life. 

Social support, or individual perceptions of available people resources [18], is generally accepted as a protective factor against caregiver burden. A recent meta-analysis of 56 studies found a moderate, negative association between social support on subjective burden (r = −0.36) among caregivers of adults and older adults [19]. Experimental findings showed that social support for caregivers can lead to a reduction in depressive symptoms over time [20]. In caregiving research, studies also connected social support and caregiving self-efficacy by showing that caregiving self-efficacy mediated the relationships of social support with some mental health outcomes such as quality of life and depression in caregivers [21,22,23,24,25].

Sources of support is an important dimension of social support, and family and friends are two of the most prominent sources of social support [26]. Given that most palliative care patients are cared for in their own homes [2], caregivers will be increasingly isolated from society [27]. Deepening the understanding of how social support affects burden of caregivers and quality of life of patients requiring palliative care is necessary. However, the associations of support from family and friends with caregiver burden are under-researched. The literature has consistently shown that support from friends was a protective factor, on the basis of the few studies reporting support from family and friends in caregivers of patients with cancer and stroke [28,29,30]. By contrast, the results for support from family were mixed: two studies reported a non-significant correlation with caregiver burden and one study found that support from family was also a protective factor [28].

Caregiving self-efficacy and sources of social support are major variables affecting the caregiving experience of caregivers in palliative care. Even though many studies among caregivers have shown caregiving self-efficacy mediated on the relationship between social support and caregiver burden [21,22,23,24,25], previous studies failed to consider that different sources of social support may moderate the relationships of caregiving self-efficacy with caregiver burden and patient’s quality of life. Therefore, whether the mediating role of caregiving self-efficacy will still be applied when support from family and friends are treated as discrete constructs is unclear. Additionally, no study has empirically examined whether caregiving self-efficacy and sources of social support of caregivers would associate with patient’s quality of life. The present study examined whether support from family and friends would associate with caregiver burden and patient’s quality of life to different extents, mediated via caregiving self-efficacy in patient–caregiver dyads with patients requiring palliative care using a structural equation modeling approach.

## 2. Materials and Methods

### 2.1. Study Design, Sample and Participants

This study is a cross-sectional study with a convenience sample of 234 Chinese patient–caregiver dyads recruited between September 2016 and May 2017 from outpatient palliative ward of one hospital and medical wards of two hospitals in Hong Kong. In this study, we adopted the WHO definition that palliative care is appropriate throughout an illness, regardless of disease stage or prognosis, rather than focusing on a later stage of disease trajectory [31]. Patients with palliative care needs were measured by NECPAL CCOMS-ICO© Tool Version 1.0 [32] along with their primary non-paid caregivers. Inclusion criteria for patients were Chinese people who were (a) 18 years or older, (b) medically stable, (c) able to communicate in Chinese, (d) primarily living at home, (e) mentally competent at the time of recruitment, and (f) had a Mini-Mental State Examination (MMSE) score > 10. Inclusion criteria for their caregivers were Chinese who were (a) 18 years or old, (b) taking care of the patient over the past 3 months, and (c) a non-paid caregiver as suggested by the patient.

### 2.2. Procedure

The study was approved by the ethical committees of the study hospitals (CREC2015.474 and VW16-105). Trained research assistants (RAs) approached potential patients and asked eligible patients to nominate a primary caregiver. They then further screened the nominated caregivers for eligibility. After obtaining written consent from the patient and the caregiver, the RA administered the questionnaires to the patient and the caregiver separately.

### 2.3. Measures

Two instruments were administered to the patients to measure their quality of life and physical functioning, and three instruments were administered to the caregivers to measure their burden, caregiving self-efficacy, and perceived social support, respectively.

Patient’s quality of life was measured by the Chinese Hong Kong version of the McGill Quality of Life Questionnaire (MQOL-HK) [33]. The MQOL-HK consists of 18 items measuring five domains, namely, (1) physical (five items), (2) psychological (six items), (3) existential well-being (four items), (4) support (two items), and (5) sex (one item). The possible range of the five domains was 0–10, with a higher score indicating better quality of life. Cronbach’s alpha values of the four domains in the current sample were 0.67 for physical component, 0.85 for psychological component, 0.82 for existential well-being, and 0.73 for support subscales.

Patient’s physical functioning was measured using the 10-item Chinese version of the modified Barthel Index (MBI) [34]. The total MBI score was computed by summing the scores of the 10 items with a possible range of 0–100, in which higher scores indicated higher independence (Cronbach’s alpha = 0.88).

Caregiver burden was measured by the Chinese version of the 13-item Caregiver Strain Index (CSI) [35]. The CSI has been widely used in studies on the burdens of caregivers of patients with chronic illnesses. A total score of CSI, with a possible range of 0–13, was computed by summing up the scores of the items, in which a higher score indicated a greater level of burden. The CSI has been shown to have good psychometric properties in a sample of caregivers in the community [36]. The Cronbach’s alpha value of the CSI in the present study was 0.84.

Caregiving self-efficacy was assessed using the 18-item modified Chinese version of the CGI (C-CGI-18) [37] adapted from the Caregiver Inventory [38] that measured caregiving self-efficacy. The C-CGI-18 has three subscales: (1) care of the care recipient (seven items) measuring positive aspects of caregiving in providing good care to the recipient and noticing good moments of caregiving; (2) managing information and self-care (seven items); measuring, seeking and understanding the medical information; and dealing with negative aspects of caregiving on oneself; and (3) managing emotional interaction with care recipient (four items) reflecting the ability in interacting actively with patients in dealing with emotional situations. The possible range of the subscale scores was 1–9, with higher scores indicating higher levels of caregiving self-efficacy. In the current sample, acceptable Cronbach’s alpha values of these subscales were in the range 0.85–0.90.

Sources of social support was measured by the Chinese version of the Multidimensional Scale of Perceived Social Support (C-MSPSS), which covers three sources of social support from family, friends, and significant others [39]. The C-MSPSS consists of 12 items which are rated on a seven-point Likert scale ranging from 1 = “strongly disagree” to 7 = “strongly agree.” A higher score indicates a higher level of perceived social support received from each source. We only used two subscales, namely, family support and friend support, in the current study. Their Cronbach’s alpha values were 0.876 and 0.888, respectively.

Demographic variables, including age, gender, educational level, marital status, and perceived financial status of caregivers and patients, were collected. Perceived financial status was measured using an item (very deficient (1) to very good (5)). Caregivers were asked to indicate their relationship with the patients, perceived good health status (very poor (1) to very good (5)) and whether a domestic helper was employed in taking care of the patient.

### 2.4. Data Analysis

**Figure 1:**
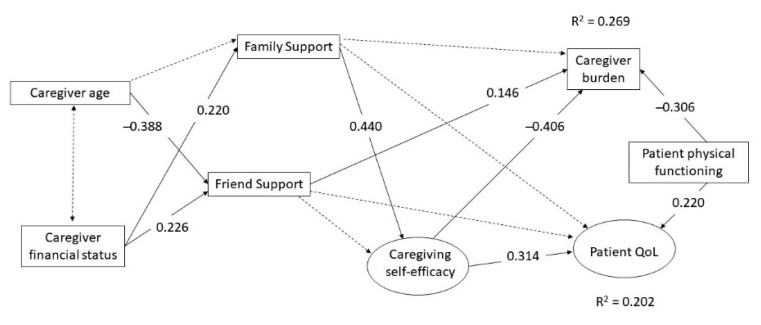
Hypothesized model linking source of social support, caregiving self-efficacy, caregiver burden, and patient’s quality of life. Insignificant paths are in dashed line and were eliminated in the final model.

Descriptive statistics to summarize the demographic data of the caregivers and the patients and to calculate the means and SDs of the survey scale scores. Structural equation modeling approach (SEM) was used to test the associations of family and friend support with caregiver burden and patient’s quality of life via the mediating factor caregiving self-efficacy. In the hypothesized model (Figure 1), caregiving self-efficacy and patient’s quality of life were latent variables with their subscales as indicators respectively. Following the terminology commonly used in SEM, a direct effect represents the effect of an independent variable on a dependent variable, an indirect effect represents the effect of an independent variable on a dependent variable through a mediating variable, and the total effect is the summation of the direct and indirect effects on the dependent variable (p. 325) [40]. The direct, indirect and total effects among the variables in our hypothesized model are dictated empirically-based suppositions that are derived from the literature. Family and friend support have direct effects and indirect effects via caregiving self-efficacy on caregiver burden and patient’s quality of life separately. Caregiver burden correlates with patient’s quality life. Statistically significant relationships with family and/or friend support were observed in variables including caregiver’s age and financial status in the current sample, so they were included as covariates in the model. Correlations were assumed between covariates, whereas direct effects were assumed between covariates and the key variables concerned. In addition, patient’s physical functioning (MBI) correlated significantly with caregiver burden and patient’s quality of life but patient’s cognitive functioning (MMSE) showed non-significance. Hence, only MBI was assumed to have direct effects on caregiver burden and patient’s quality of life. Before fitting the hypothesized model, we first used confirmatory factor analysis to assess the goodness-of-fit of the measurement model of the hypothesized model to the data by setting all the directional paths among the variables as co-directional paths in Figure 1. Among the 234 patient-caregiver dyads, 225 (96.2%) dyads provided complete data in all 14 variables, and they were included in the current analysis. A sample size of 223 is sufficient to detect an effect size 0.2 with 2 latent variables and 14 observed variables at a 5% significance level and 80% power [41].

The EQS 6.0 package (Multivariate Software Inc, Encino, CA, USA)) with the robust maximum likelihood estimation procedure [42,43] was used to estimate the models. Multiple criteria, including relative fit and absolute misfit indices, were considered when assessing the model fit. The standardized root mean squared residual (SRMR) and the absolute misfit indices represent the robust root mean square error of approximation (R-RMSEA). The Robust Comparative Fit Index (R-CFI) was used to compute the relative goodness-of-fit index. A model with an SRMR of <0.08, an R-RMSEA of <0.08 and an R-CFI of >0.9 is considered to have an acceptable fit to the data [44]. The direct effects are estimated by the regression coefficients and the indirect effects are estimated based on Sobel test in EQS [42]. A statistical significance level of 5% was set for the estimated paths in the proposed model. In addition, based on multivariate Lagrange multiplier tests, post-hoc modifications to the proposed model were made to add new paths. Wald tests were used to delete paths provided by EQS and for the paths’ theoretical plausibility, allowing us to create closely related models with a better fit to the data. To obtain a more parsimonious model, a final model was then estimated with only statistically significant paths retained. The corrected Satorra–Bentler robust χ^2^ difference (∆R-χ^2^) test was used to compare the goodness of fit between the initial and final models. All statistical tests were considered significant at *p*-value < 0.05. SPSS 25.0 (IBM, Armonk, NY, USA) was used for all statistical tests except for the SEM analyses.

## 3. Results

### 3.1. Participant Characteristics

Table 1 summarizes the demographic and health-related characteristics of patients and caregivers. The mean age of patients was 77 years (SD = 10.4). A total of 40.4% were male and 42.2% did not have any formal education. Of the patients, 51% had a leading diagnosis of heart disease and 19.1% had cancer, and 27.6% were receiving regular outpatient palliative care at the hospital. The patients reported a high mean level of independence (MBI). The mean age of the caregivers was 57.2 years (SD = 14.7), 34.3% were male, 67.9% had secondary education or above, and 50.9% were the children of the patients. Approximately 25% of the caregivers had a domestic helper to help in taking care of the patient, and 10.6% of the patients were receiving long-term care services. Only 20.3% of the patients and 13.4% of the caregivers had sufficient financial resources to support their daily expenses.

Means, standard deviations, and ranges were computed for the major variables in the study model (Table 2). Overall speaking, the caregivers in the sample had a low mean level in caregiver burden, moderate to high mean levels in the three subscales of caregiving self-efficacy, and moderate mean levels of perceived social support from family and friends. For the patients, they reported a high mean level of physical functioning and moderate to high mean levels in the five domains of quality of life.

### 3.2. Path Analysis Results

The covariance matrix of the 14 variables was submitted to EQS to estimate the parameters and test the plausibility of the measurement model and the hypothesized model. Satisfactory model fits to the data were obtained for the measurement model (R-χ^2^ = 124.470, d.f. = 67, SRMR = 0.080, R-RMSEA = 0.062 and R-CFI = 0.913) as well as the initial hypothesized model (R-χ^2^ = 115.0182, d.f. = 67, SRMR = 0.067, R-RMSEA = 0.057 and R-CFI = 0.928). After eliminating six insignificant paths based on the Wald tests from the initial hypothesized model, the final model provided a fit (R-χ^2^ = 122.2938, d.f. = 73, SRMR = 0.070, R-RMSEA = 0.055 and R-CFI = 0.926) with the data as good as the initial model (∆R-χ^2^ = 6.95, d.f. = 6, *p*-value = 0.326). The results of the standardized parameter estimates for the final model are also shown in Figure 1. Friend support has a significant positive path to caregiver burden only. On the other hand, family support exhibited a different pattern that had a significant positive path to caregiving self-efficacy, which in turn had a significant negative path to caregiver burden and a significant positive path to patient’s quality of life. For the covariates, caregiver’s financial status had significant positive paths to both family and friend support, whereas caregiver’s age had a significant negative path to friend support only. Patient’s physical functioning has a significant negative path to caregiver burden and a positive path to patient’s quality of life.

The standardized direct, indirect, and total effects of the study variables on caregiver burden and patient’s quality of life are presented in Table 3. The results showed that family support had a significant negative indirect effect on caregiver burden and a significant positive indirect effect on patient’s quality of life through caregiving self-efficacy, whereas friend support had a significant positive direct effect on caregiver burden but a minimal effect, if any, on patient’s quality of life. For covariates, caregiver’s age had a significant and negative indirect effect on caregiver burden. Caregiver’s financial status had a negative indirect effect on caregiver burden and a positive indirect effect on patient’s quality of life, but both the effects were non-significant. 

## 4. Discussion

The objective of this study was to address important gaps in the literature by investigating the differential magnitude of associations of social support from family and friends on caregiver burden and patient’s quality of life, mediated by caregiving self-efficacy in Chinese patient–caregiver dyads of patients with palliative care needs. Mediation analysis results suggested that family support correlated positively, mediated through caregiving self-efficacy, with caregiver burden and patient’s quality of life, whereas friend support has a negative direct effect on caregiver burden only. The results implied that caregivers perceived to be having a higher level of family support tended to have a higher caregiving self-efficacy, which in turn is associated with a lower level of burden and a higher quality of life in the patient. Friend support, by contrast, was a risk factor of caregiver burden and was not associated with patient’s quality of life among Chinese patients with palliative care needs and their caregivers.

This study extended knowledge by performing mediation analysis to elucidate the relationships of perceived family and friend support and caregiving self-efficacy with caregiver burden and patient’s quality of life in the palliative care setting. For the relationships between social support and caregiver burden, contrary to a previous study in Chinese [30], our study found that family support, and not friend support, was a protective factor against caregiver burden through the mediating effect of caregiving self-efficacy. By contrast, friend support was associated positively with caregiver burden. The differences in the directions of relationships of family and friend support with caregiver burden could be explained by the characteristics of the family-centered social support system used by Chinese people. In this social support system, emotional, financial, and instrumental support are given, and family members are the major providers. Two additional cultural characteristics, namely, shame and harmony, shape the behaviors of giving and receiving help [45]. Han’s study [30] focused on stroke survivors within 6 months who were severely dependent and required highly demanding care. As predicted by the family-centered social support system, a high level of family support with small variation was observed, possibly contributing to the absence of a relationship between family support and caregiver burden. By contrast, the patients’ severe situation was obvious. Thus, it would be appropriate for the caregivers to receive help from someone outside the family support system without feeling shame, and friend support would be praised and viewed positively. Compared with the patients in Han’s study [30], the patients in our study were highly physically independent. Thus, the care needs of patients and the caregivers might not be visible. In this situation, family support might not be thought of as necessary, so emotional, financial, and instrumental support from family could lead to an increase in caregiving self-efficacy, in turn reducing caregiver burden and enhancing patient’s quality of life. However, caregivers might consider that discussing their needs openly or seeking and receiving help from friends was culturally inappropriate because receiving help from someone outside the family might lead to feelings of shame or a potential disruption of harmony in the family [27,46,47,48], thereby possibly having a negative effect on caregiver burden. The findings of the present study emphasize that sources of social support is an important dimension influencing caregiver burden and patient’s quality of life via different pathways, and the direction of associations may depend on patient’s characteristics. Nevertheless, social support is a multidimensional concept with several important aspects such as the amount, the type, the sources, the structure, and the functional properties of the social support; future studies including a finer measure of social support that covers these five important aspects could get a clearer picture of the associations among social support, caregiving self-efficacy, caregiver burden, and quality of life [26,45]. In addition, we recommend replicating the current study in patients and caregivers with different cultural backgrounds in order to explore whether cultures play an important role.

Another important finding of the study is that caregiving self-efficacy has significant associations with caregiver burden and patient’s quality of life. This finding extends the important role of caregiving self-efficacy from its association with caregiver outcomes (i.e., positive aspects of caregiving, caregiver burden, and quality of life) to patient outcomes, and quality of life in particular [49]. Previous studies postulated a strong role for caregivers’ self-efficacy in the positive aspects of caregiving [50], and the positive aspects of caregiving were important determinants of quality of care provided to the recipient [9]. Importantly, caregiving self-efficacy is amenable to be enhanced [10]. Therefore, establishing a relationship between caregiving self-efficacy and patient’s quality of life may have practical implications for caregiver interventions.

In this study, we found that caregiver burden was not associated with patient’s quality of life. This finding contradicts previous findings that reduced caregiver burden was associated with improved patient outcomes [51,52]. A possible reason for this finding is our inclusion of patients who were living at home, possibly resulting in the exclusion of overburdened caregivers of patients with high dependency because they might have already sought for a paid nursing home placement from the private sector. Exclusion of dyads of overburdened caregivers and high dependency patients in the study would have diluted the strength of the association between caregiver burden and patient’s quality of life. Alternatively, filial piety is an important value among Chinese people. Several caregivers might consider that providing care to the patient is a responsibility rather than a burden. Filial attitude was reported as a protective factor against caregiver depression in China [53]. Moreover, in samples of Chinese caregivers, positive aspects of caregiving were found to be uncorrelated with caregiver burden and role overload [11]. Previous studies did not include caregiving self-efficacy and caregiver burden in predicting patient outcomes [51,54,55]. Our study used a structural equation modeling approach to disentangle the associations of caregiver burden and caregiving self-efficacy on patient’s quality of life and showed that caregiving self-efficacy is associated with both caregiver burden and patient’s quality of life. Nevertheless, this work can be replicated with additional caregiving-related factors, such as filial piety and positive aspects in caregiving, in the analyses to examine their contributions to patient’s quality of life simultaneously in a structural model.

For covariates, we found patient’s physical functioning associated with reduced caregiver burden and increased patient’s quality of life, which is consistent with the literature [56,57]. In addition, we found caregiver’s age had a small and significant negative indirect effect on caregiver burden, which is in line with the results of a systematic review on family caregivers of elderly cancer patients in which younger age is a significant factor associated with higher caregiver burden with moderate level of evidence [56]. Caregiver’s financial status has a small and non-significant indirect effects on caregiver burden and caregiver’s financial status has a small and non-significant indirect effect on patient’s quality of life. Previous studies also reported mixed results on the relationship between financial status and burden in caregivers [58,59].

This study has several limitations. First, the findings of associations among the variables are based on a cross-sectional design, and hence, should be further examined in longitudinal studies to establish the temporal validity of any associations found. Second, as a secondary analysis of data collected for other research purposes, important caregiving-related factors, such as positive aspects in caregiving, role conflict, care strategies, emotional support to caregivers, and caregiver’s quality of life were not measured [9,60,61]. This situation precluded us from investigating independent and potential interaction effects on patient’s and caregiver’s quality of life. The strength of our study is the inclusion of a heterogeneous sample of patients with different chronic diseases, and some were not receiving palliative care although with needs, but this factor could also be a limitation. In particular, our subjects had high physical functioning scores that will definitely limit the generalization of the study findings to the population of patients receiving palliative care. In the study, we included measures to assess patient’s physical functioning and cognitive status but not depression or disease-specific factors, such as types of treatment for cancer patients that were reported to be related with quality of life [49]. This limitation could partly explain the limited explanatory power of the predictors for patient’s quality of life. In addition, we did not measure the actual tasks in caregiving for the caregiver. Some tasks such as physically turning and feeding are expected to induce more burden compared with other tasks such as cooking. The strength and the direction of the associations between the factors and the outcome variables might be different if these potential factors were available for analysis. The use of the convenience sample and the small sample size in this study certainly limited the generalizability of the findings.

## 5. Conclusions

This study illustrated the associations of caregiving self-efficacy and sources of social support with caregiver burden and patient’s quality of life in Chinese patients requiring palliative care and their caregivers. The results showed that family support had a significant negative indirect effect on caregiver burden and a significant indirect effect on patient’s quality of life through caregiving self-efficacy, whereas friend support had a significant positive direct effect on caregiver burden in this sample of participants. The inter-relationship of the four variables improves our understanding of the caregiving experience of caregivers and patients with palliative care needs. Providing social support from appropriate sources and enhancing caregiving self-efficacy may be important strategies in relieving caregiver burden and improving patient’s quality of life, but further study is needed to build up a body of evidence.

## Figures and Tables

**Table 1:** Characteristics of primary caregivers (*n* = 225).

Characteristics	Patients	Caregivers
Age, Mean ± SD	76.8 ± 10.4	57.1 ± 14.6
Male, *n* (%)	91 (40.4%)	79 (35.1%)
Married/Cohabitation, *n* (%)	138 (61.3%)	187 (83.1%)
Educational level, *n* (%)		
No formal education	95 (42.2%)	20 (8.9%)
Primary education	72 (32.0%)	50 (22.2%)
Secondary education or above	56 (24.8%)	154 (68.4%)
Missing	2 (0.9%)	1 (0.4%)
Perceived financial status, *n* (%)		
Completely not enough	14 (6.2%)	19 (8.4%)
Not enough	45 (20.0%)	39 (17.3%)
Neutral	119 (52.9%)	138 (61.3%)
Enough	34 (15.1%)	22 (9.8%)
Completely enough	10 (4.4%)	7 (3.1%)
Missing	3 (1.3%)	
Receiving regular outpatient palliative care at hospital	62 (27.6%)	
Leading class of diagnosis, *n* (%)		
Diabetes	40 (17.8%)	
Heart diseases	115 (51.1%)	
Neurological diseases	6 (2.7%)	
Kidney diseases	7 (3.1%)	
Cancer	43 (19.1%)	
Lung diseases	11 (4.9%)	
Bone diseases	3 (1.3%)	
MBI, Mean ± SD	85.3 ± 19.5	
MMSE, Mean ± SD	22.1 ± 5.2	
Relationship with the patient, *n* (%)		
Child		116 (51.6%)
Spouse		83 (36.9%)
Others		26 (11.6%)
Perceived health status, Mean ± SD		3.28 ± 0.97
Has a domestic helper, *n* (%)		57 (25.3%)

Note: MBI = Modified Barthel Index; MMSE = Mini-Mental State Examination.

**Table 2:** Descriptive statistics of outcome measures in patients and their caregivers (*n* = 225).

Participants	Variable	Possible Range	Mean ± SD	Median (Min, Max)
Caregivers	Caregiver burden	0–13	5.81 ± 3.8	6.0 (0, 13)
	Caregiving self-efficacy			
	Care of the care recipient	1–9	7.08 ± 1.37	7.1 (1.4, 9)
	Managing information and self-care	1–9	6.37 ± 1.51	6.4 (1, 9)
	Managing emotional interaction with care Recipient	1–9	6.46 ± 1.71	6.5 (1, 9)
	Family Support	1–7	5.11 ± 1.16	5.3 (1.3, 7)
	Friend Support	1–7	4.09 ± 1.33	4.3 (1, 7)
Patients	Physical functioning	0–100	84.93 ± 19.71	92.0 (0, 100)
	Quality of life			
	Physical	0–10	5.21 ± 1.83	5.0 (1.3, 10)
	Psychological	0–10	7.96 ± 2.16	8.7 (1, 10)
	Existence	0–10	6.24 ± 2.01	6.3 (0, 10)
	Support	0–10	7.08 ± 1.87	7.0 (0, 10)
	Sex	0–10	8.40 ± 3.00	10.0 (0,10)

**Table 3:** Standardized direct, indirect and total effects of variables on caregiver burden and patient’s quality of life.

Path	Direct Effect	Indirect Effect	Total Effect
To caregiver burden			
Family support	-	−0.162 *	−0.162 *
Friend support	0.166 *	-	0.166 *
Caregiving self-efficacy	−0.406 *	-	−0.406 *
Caregiver’s age	-	−0.064 *	−0.054
Caregiver’s financial status	-	0.002	−0.007
Patient’s physical functioning	−0.306 *	-	−0.306 *
To patient’s quality of life			
Family support	-	0.158 *	0.158 *
Friend support	-	-	-
Caregiving self-efficacy	0.314 *	0.314 *	0.314 *
Caregiver’s age	-	-	-
Caregiver’s financial status	-	0.035	0.071
Patient’s physical functioning	0.220 *	-	0.220 *

* *p*-value < 0.05.
